# The revised role of TGF-β in aortic aneurysms in Marfan syndrome

**DOI:** 10.1007/s12471-014-0622-0

**Published:** 2014-10-24

**Authors:** R. Franken, T. Radonic, A. W. den Hartog, M. Groenink, G. Pals, M. van Eijk, R. Lutter, B. J. M. Mulder, A. H. Zwinderman, V. de Waard, COMPARE study group

**Affiliations:** 1 Department of Cardiology, Academic Medical Centre Amsterdam, Amsterdam, the Netherlands; 2 Interuniversity Cardiology Institute of the Netherlands, Utrecht, the Netherlands; 3 Department of Pathology, VU University Medical Centre Amsterdam, Amsterdam, the Netherlands; 4 Department of Radiology, Academic Medical Centre Amsterdam, Amsterdam, the Netherlands; 5 Department of Clinical Genetics and DNA Diagnostics, VU University Medical Centre Amsterdam, Amsterdam, the Netherlands; 6 Department of Medical Biochemistry, Academic Medical Centre Amsterdam, Meibergdreef 15, Post-box 22660, 1105 AZ Amsterdam, the Netherlands; 7 Department of Pulmonology and Experimental Immunology, Academic Medical Centre Amsterdam, Amsterdam, the Netherlands; 8 Durrer Cardiogenetic Research Centre Utrecht, Utrecht, the Netherlands

**Keywords:** Marfan syndrome, Losartan, Therapeutic effect, Transforming growth factor-β (TGF-β), Angiotensin II

## Abstract

**Background:**

Recently, we demonstrated that losartan reduced the aortic root dilatation rate (AoDR) in adults with Marfan syndrome (MFS); however, responsiveness was diverse. The aim was to determine the role of transforming growth factor-β (TGF-β) as therapeutic biomarker for effectiveness of losartan on AoDR.

**Methods:**

Baseline plasma TGF-β levels of 22 healthy controls and 99 MFS patients, and TGF-β levels after 1 month of losartan treatment in 42 MFS patients were measured. AoDR was assessed by magnetic resonance imaging at baseline and after 3 years of follow-up.

**Results:**

Patients with MFS had higher TGF-β levels compared with healthy controls (121 pg/ml versus 54 pg/mL, *p* = 0.006). After 1 month of therapy, losartan normalised the TGF-β level in 15 patients (36%); the other 27 patients (64%) showed a significant increase of TGF-β. After 3 years of losartan therapy, patients with a decrease in TGF-β had significantly higher AoDR compared with patients with increased TGF-β (1.5 mm/3 years versus 0.5 mm/3 years, *p* = 0.04). Patients showing a decrease in TGF-β after losartan therapy had significantly elevated baseline TGF-β levels compared with patients with increased TGF-β (189 pg/ml versus 94 pg/ml, *p* = 0.05).

**Conclusion:**

Patients responding to losartan therapy with a reduction of the plasma TGF-β level had higher baseline TGF-β levels and a higher AoDR. Most likely, TGF-β levels may be considered to be a readout of the disease state of the aorta. We propose that increased angiotensin II is the initiator of aorta dilatation and is responsible for increased TGF-β levels in MFS. The concept of TGF-β as initiator of aortic dilatation in MFS patients should be nuanced.

## Introduction

Aortic root dilatation is a hazardous complication in patients with Marfan syndrome (MFS), an heritable connective tissue disorder equally prevalent all over the world [1, 2]. MFS is caused by mutations in the *FBN1* gene [3]. These mutations induce abnormal or deficient fibrillin-1 protein affecting the structural integrity of the vascular extracellular matrix, and have been described to enhance the release of transforming growth factor-β (TGF-β) [4]. We have shown that plasma TGF-β was indeed elevated in MFS patients, which correlated to increased aortic root diameter and aortic root dilatation rate (AoDR) [5].

Current treatment comprises prophylactic aortic root replacement and β-blocker therapy, which has significantly improved the life expectancy of patients with MFS. However, cardiovascular complications remain a problem [6, 7]. In a MFS mouse model, losartan was superior to β-blocker therapy in decreasing AoDR. This losartan effect was attributed to reduced TGF-β expression, which was mimicked by treatment with neutralising TGF-β antibodies [8].

These findings in mice have resulted in the initiation of multiple studies assessing the effect of losartan on AoDR in MFS patients. Recently, we demonstrated that losartan reduced AoDR in a randomised and prospective cohort of adult patients with MFS (COMPARE trial) [9, 10]. In addition, a small study in 20 children revealed beneficial effects of losartan on AoDR. [11, 12] However, the responsiveness to losartan treatment was diverse, which may depend on variability in expression and release of TGF-β. In order to determine the role of TGF-β as a therapeutic biomarker for the effectiveness of losartan therapy on AoDR, we performed a sub-study of the COMPARE trial and revealed that TGF-β is an indirect effector of aortic dilatation.

## Effect of losartan therapy on plasma TGF-β levels

**Fig. 1:**
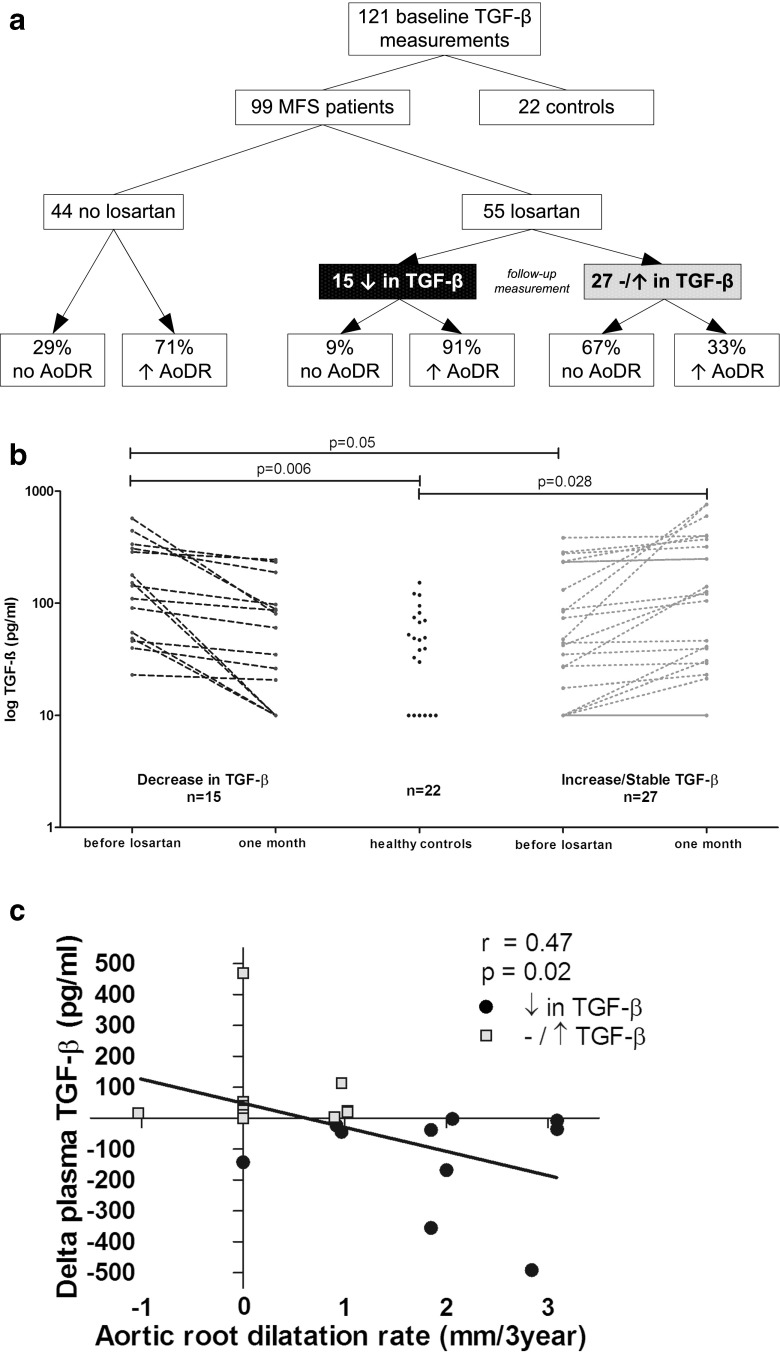
**a** Flowchart shows an overview of Marfan syndrome patients and controls. Plasma TGF-β was analysed by ELISA (R&D Systems). **b** In 15 of the 42 MFS patients losartan normalised plasma TGF-β levels to that of controls. In 27 of MFS patients no reduction of plasma TGF-β was observed. **c** Decrease in TGF-β level is associated with an increase in AoDR in patients using losartan therapy (r = 0.47, *p* = 0.02). Linear regression analysis was used. AoDR = Aortic root dilatation rate; TGF-β = transforming growth factor β

For this sub-study we measured baseline TGF-β levels of 99 MFS patients, all monitored by the Academic Medical Centre Amsterdam, of whom 55 were randomised to 100 mg losartan and 44 to no losartan. In 42 patients on losartan therapy, plasma TGF-β levels were also assessed after 1 month of treatment. We recruited 22 ‘healthy controls’ in whom MFS was definitely ruled out (Fig. 1a). In order to determine AoDR, MFS patients underwent magnetic resonance imaging of the aorta at baseline and after 3 years of follow-up.

Despite inter-laboratory variations in TGF-β measurements throughout the medical world, our TGF-β measurements show that MFS patients had significantly higher TGF-β levels compared with healthy controls (121 pg/ml versus 54 pg/mL, *p* = 0.006), which is in line with our previous results [2]. Surprisingly, 1 month of losartan therapy did not reduce circulating TGF-β levels (101 pg/mL; 95% CI: −27:229 pg/mL; *p* = 0.12) (Fig. 1b). TGF-β levels were only normalised to the control level in 15 of the 42 patients (*p* = 0.26). In the remaining 27 patients TGF-β did not decrease after one-month losartan therapy, and showed a significantly higher TGF-β compared with the healthy controls (292 pg/ml versus 54 pg/mL, *p* = 0.028). Unexpectedly, after 3 years of losartan therapy, patients with a decrease in TGF-β had a significantly higher AoDR compared with patients without reduced TGF-β levels (1.5 ± 0.8 mm/3 years versus 0.5 ± 1.2 mm/3 years, respectively; *p* = 0.04). In addition, 91% of the patients with a decrease in TGF-β showed a significant increase in AoDR despite losartan therapy, compared with 33% of the patients with increased/stable TGF-β levels after losartan (*p* = 0.013) (Fig. 1a,b). In order to explain these unexpected results, we compared baseline characteristics between these groups. Patient groups were comparable, with the exception of baseline TGF-β levels (Table 1). Patients showing a decrease in TGF-β after losartan therapy had significantly elevated baseline TGF-β levels compared with patients who did not show this decrease (189 ± 166 pg/ml versus 94 ± 113 pg/ml, *p* = 0.05) (Fig. 1b). Interestingly, we found a linear association between the change in TGF-β level and the increase in AoDR in patients using losartan therapy (r = 0.47, *p* = 0.02) (Fig.1c). This effect could not be ascribed to β-blocker therapy, because β-blocker therapy +/− losartan therapy was not different at any level (data not shown).

**Table 1:** Patient characteristics according to the change in TGF-β after losartan therapy

	Decreased TGF-β	Increased/stable TGF-β	
MFS patients with:	(*n* = 15)	(*n* = 27)	*p*-value
*Clinical features*			
Mean age (SD)	38 (10)	35 (11)	
Sex (male)	7 (47)	18 (67)	
Baseline TGF-β level*	189 (166)	94 (113)	0.05
Cardiovascular			
AoR dilatation	15 (100)	22 (81)	
Mean AoR diameter (SD)	45 (4)	43 (6)	
AoR operation	4 (27)	9 (33)	
Mean age AoR operation (SD)	31 (11)	31 (15)	
MV prolapse	12 (80)	17 (63)	
MV repair	1 (7)	3 (11)	
FH of dissection	6 (40)	12 (44)	
Dilatation of distal aorta	2 (13)	3 (11)	
β-blocker	10 (67)	21 (78)	
dosage >100 mg	5 (33)	14 (52)	
dosage <100 mg	5 (33)	7 (26)	

Values are given in absolute numbers (percentage) if not otherwise indicated

SD: standard deviation; AoR: aortic root; MV: mitral valve; FH: family history

## Revised role of TGF-β in aortic aneurysms in Marfan syndrome

This sub-study highlights a paradoxical topic of TGF-β in the pathogenesis of MFS. Although 3 years of losartan therapy reduces the overall AoDR in MFS patients [9], only one-third of MFS patients responded by a reduction of plasma TGF-β after 1 month of losartan therapy. These responders had elevated baseline TGF-β levels and an increase in AoDR after 3 years, despite losartan therapy. Considering treatment with 100 mg losartan is a sufficient dose to reduce AoDR in MFS [9], and assuming that one-month treatment is sufficient to initiate a TGF-β response, we have three possible explanations for our findings.

The first explanation for the fact that losartan did not reduce overall TGF-β levels is that TGF-β is a readout of the diseased state of the aorta. Losartan did reduce TGF-β levels in a subgroup of MFS patients, yet these patients revealed a higher AoDR after 3 years of therapy. This increase in AoDR may be explained by the elevated baseline TGF-β levels and the slightly, but non-significant larger aortic root dimension at baseline (45 ± 4 mm versus 43 ± 6 mm, *p* = 0.215). Both factors are associated with an increase in AoDR. [5] These results suggest that elevated plasma TGF-β is a marker for aortic damage, such as fibrosis, rather than the initial cause of aortic dilatation.

A second explanation for the variable response of TGF-β to losartan therapy comprises the multitude of *FBN1* mutations. At present, more than 2900 different mutations have been described in the Universal Mutation Database [13]. Most *FBN1* mutations result in expression of mutated fibrillin-1 proteins, which are improperly folded. The abnormal fibrillin-1 protein may have a dominant-negative effect on the structure of the extracellular fibrillin network when it interacts with normal fibrillin-1 protein of the non-mutated allele and other extracellular matrix proteins. Both the strength of the fibrillin-1 matrix may be changed as well as the release of TGF-β that is captured in the fibrillin-1 network. Mutations in one of the seven TGF-β binding protein-like (TB) domains of the *FBN1* gene may especially alter TGF-β levels.

Other *FBN1* mutations will lead to reduced fibrillin-1 protein levels as a result of deletion of the entire *FBN1* gene on one allele [14] or for example upon deletion of the first exon of *FBN1* (such that the mRNA is not translated), causing ‘haploinsufficiency’. The reduced level of normal fibrillin-1 protein presumably results in a thinner fibrillin-1 matrix in the vasculature and thus in reduced aortic wall strength. In such patients, angiotensin II (AngII) activation may be increased to maintain normal blood pressure. The AngII-mediated signalling cascade is a common inducer of TGF-β production in the vessel wall and thus involved in the increased plasma TGF-β levels in these patients. Blocking the AngII receptor-1 (AT1) with losartan will diminish TGF-β production as well as other AngII-mediated detrimental processes in the vessel wall such as blood pressure increase, enhanced pro-inflammatory responses, myofibroblast differentiation and reactive oxygen species (ROS) generation. Therefore, we hypothesise that in patients with an *FBN1* mutation leading to haploinsufficiency, the effect of losartan on aortic dilatation is better compared with patients with a dominant-negative mutation. We anticipate that genetics plays a critical role in the TGF-β response to losartan [15].

A third explanation for the variable TGF-β response to losartan therapy comprises variation in the abundance of AT1 expression or activity, or by polymorphisms in the renin-angiotensin-aldosterone system (RAAS). Increased AngII-mediated signalling as a result of this type of polymorphisms will coincide with increased TGF-β production. A number of polymorphisms have been identified in RAAS that are associated with aneurysms. For example, the ACE deletion/insertion polymorphism is significantly associated with an increased risk for thoracic aortic aneurysm formation [16].

## Angiotensin II as a cause of aortic dilatation?

**Fig. 2:**
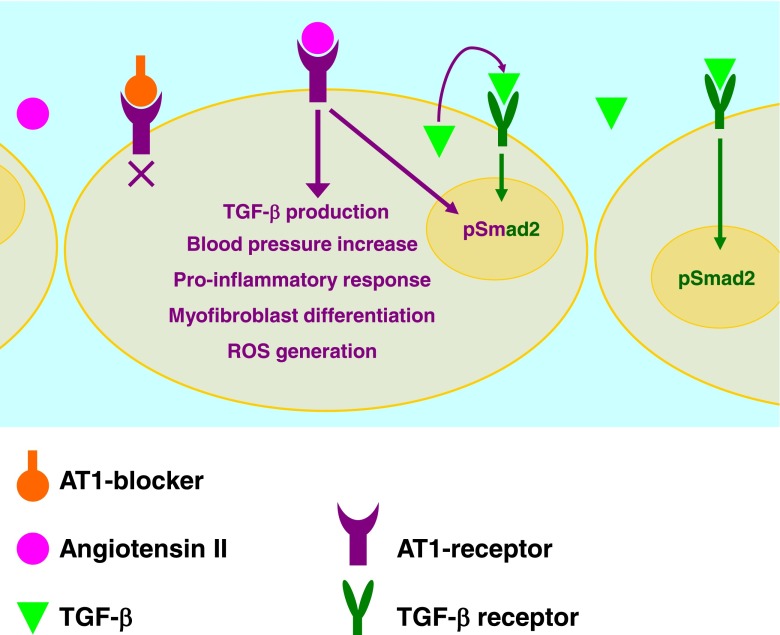
Schematic overview of proposed mechanism involving AngII- and TGF-β-mediated signalling in aortic dilatation in MFS. AngII induces a number of detrimental processes via AT1 when (chronically) elevated. AngII directly activates Smad2 (pSmad2) and increases TGF-β production, which can be secreted and subsequently binds to its cell surface receptor and thereby increases Smad2 activation further. Losartan blocks AT1 and thus inhibits AngII-mediated signalling including Smad2 activation, TGF-β production, blood pressure increase, pro-inflammatory responses, myofibroblast differentiation and ROS generation. AngII = angiotensin II; AT1 = angiotensin II receptor type 1; pSmad2 = phosphorylated Smad2; ROS = reactive oxygen species; TGF-β = transforming growth factor β

In MFS mice, direct inhibition of TGF-β was effective against AoDR. [8] However, in most studies TGF-β signalling has been extrapolated from the abundance of Smad2 activation (pSmad2) in the dilated aortic tissue [17]. Smad signalling is best known from its role in the TGF-β-induced signalling cascade, where it transfers the extracellular TGF-β signal to the nucleus to act as a transcription factor and to regulate gene expression [18]. Before birth, TGF-β is essential for the development of the cardiovascular system [19]. In later life, TGF-β is expressed in reaction to injury, mediating a fibrotic response for repair. This accentuates the possibility that TGF-β is a result and not the cause of aortic damage. Interestingly, AngII can induce Smad2 activation directly through its receptor AT1 [20, 21], as well as indirectly by enhancing TGF-β expression (Fig. 2). Thus, increased levels of TGF-β and pSmad2 levels in MFS may both result from increased AngII-mediated signalling. When AngII is considered a primary cause of aortic disease in MFS, other detrimental AngII-mediated pathways may be responsible for initiation of arterial damage (Fig. 2). Excessive TGF-β signalling subsequently leads to secondary disease progression.

In mice, chronic infusion of AngII is known to affect the integrity of the vasculature resulting in aneurysms in the ascending and descending aorta [22, 23]. We propose that the beneficial effect of losartan on AoDR in MFS mice and patients [5, 6, 8] is obtained through inhibition of the unfavourable AngII-mediated signalling cascades, involving TGF-β synthesis and pSmad2 signalling, blood pressure increase, myofibroblast differentiation, ROS generation and pro-inflammatory responses [22, 24].

## Limitations

We wish to emphasise that the power of our study was limited, therefore a prospective trial with a larger patient cohort is needed to confirm our results. Furthermore, TGF-β levels are known to vary between different laboratories. In order to prevent these variations, we collected plasma samples and performed all TGF-β measurements in the same laboratory at the same time. Finally, it would have been interesting to have the follow-up TGF-β measurements after 3 years of losartan therapy. Despite these study limitations, our results are in line with our previous results [5].

## Conclusion

In conclusion, the variable effect of losartan on plasma TGF-β levels probably reflects, at least in part, the heterogeneity in *FBN1* mutations or RAAS modifiers. We showed that MFS patients who responded with a decrease in plasma TGF-β level during losartan therapy had higher baseline TGF-β levels. Most likely, TGF-β levels may be considered to be a readout of the disease state of the aorta. Significantly, the effectiveness of losartan on the AoDR in MFS patients [6, 8] proves that Ang II-mediated signalling is crucial in the vascular pathology of MFS. We propose that increased AngII signalling is the initiator of aorta dilatation and is responsible for the increased TGF-β levels in MFS. The concept of TGF-β as the initiator of aortic dilatation in MFS patients should be nuanced now it is clear that AngII-mediated signalling is instructive and affects more than just TGF-β levels.

## Abbreviations

AngII: Angiotensin II AngII is a peptide hormone that causes vasoconstriction and a subsequent increase in blood pressure AngII is part of the RAAS
AoDR: Aortic root dilatation rate AoDR is the rate the aortic root dilates in grown-up MFS patients in mm/year
AT1: Angiotensin II type 1 receptor AT1 is a receptor with AngII as its ligand and is subsequently responsible for the signal transduction leading to vasoconstriction. AT1 is part of the RAAS and is blocked by AT1-blockers, including losartan
MFS: Marfan syndrome MFS is a connective tissue disorder and diagnosed according to the Ghent criteria of 2010 [25]
RAAS: Renin-angiotensin-aldosterone system RAAS is a hormone system that regulates blood pressure and water (fluid) balance
ROS: reactive oxygen species ROS are a natural by-product of the oxygen metabolism. ROS levels can increase dramatically during times of environmental stress, and may subsequently damage cell structures
pSmad2: phosphorylated Smad2 pSmad2 is the activated form of Smad2, a transducer protein that mediates the signal of TGF- β
TGF-β: transforming growth factor-β TGF-β is a multifunctional peptide that controls proliferation, differentiation and other functions in many cell types, and is thought to be the detrimental cause of MFS
